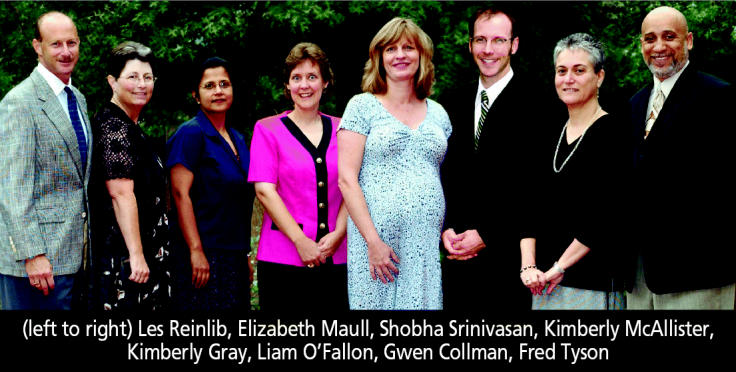# Susceptibility and Population Health Branch

**Published:** 2004-11

The Susceptibility and Population Health Branch (SPHB) plans and administers extramural programs that study the complex interplay of all factors in the environment. The overarching theme of susceptibility, broadly determined by genetic, behavioral, and sociocultural factors, is present in all SPHB programs. The hallmark of the SPHB is the creation of unique research programs that brings together diverse groups of people—scientists of many disciplines, community and advocacy members, and health care professionals—to work on research that translates basic science into effects on human populations and unique interventions to protect public health. The program areas of science education and community outreach are represented, as well, in order to disseminate scientific research to the public. Involving and informing the general public is key to the success of the SPHB programs.

The SPHB manages a variety of center programs, including the NIEHS Core Centers, along with other projects in a number of subject areas:

- NIEHS Core Centers—These centers provide infrastructural support to universities in order to promote multidisciplinary research and outreach in the environmental health sciences.
- Environmental Genome Project—This project comprises discovery and resequencing of single-nucleotide polymorphisms, the Comparative Mouse Genomics Centers Consortium, and programs in molecular epidemiology and bioethics.
- Centers for Children’s Environmental Health and Disease Prevention Research—These centers explore topics such as asthma, autism, learning, growth, and development.
- Environmental justice/community-based participatory research—This program area covers issues particular to subgroups of the population who may be disproportionately exposed, who may be disadvantaged, or who may experience disparities in disease prevalence.
- Breast Cancer and the Environment Research Centers—These centers conduct research on development of the mammary gland across the life span and effects of environmental exposures that may impact development.
- Centers for Oceans and Human Health—These centers conduct research in marine sciences that will lead to improved public health.

## SPHB Staff

**Gwen Collman, PhD—CHIEF** collman@niehs.nih.gov

NIEHS Core Centers

**Kimberly Gray, PhD—PROGRAM ADMINISTRATOR** gray6@niehs.nih.gov

Epidemiology, exposure assessment, child health

**Elizabeth Maull, PhD—PROGRAM ADMINISTRATOR** maull@niehs.nih.gov

Botanical research, breast cancer

**Kimberly McAllister, PhD—PROGRAM ADMINISTRATOR** mcallis2@niehs.nih.gov

Genetic susceptibility, molecular epidemiology

**Les Reinlib, PhD—PROGRAM ADMINISTRATOR** reinlib@niehs.nih.gov

Breast cancer, carcinogenesis, DNA repair

**Shobha Srinivasan, PhD—PROGRAM ADMINISTRATOR** sriniva2@niehs.nih.gov

Health disparities, community-based research

**Fred Tyson, PhD—PROGRAM ADMINISTRATOR** tyson2@niehs.nih.gov

Oceans, health disparities, genomics, Advanced Research Cooperation in Environmental Health

**Liam O’Fallon, MA—PROGRAM ANALYST** ofallon@niehs.nih.gov

Primary and secondary school science education, community outreach and education

## Figures and Tables

**Figure:**